# Ligustilide Improves Cognitive Impairment via Regulating the SIRT1/IRE1*α*/XBP1s/CHOP Pathway in Vascular Dementia Rats

**DOI:** 10.1155/2022/6664990

**Published:** 2022-08-16

**Authors:** Dong Peng, Yi-Xue Wang, Tian-Hua Huang, Dan Luo, Li-Jun Qiao, Qi Wang, Li Guan, Ye-Feng Cai, Shi-Jie Zhang

**Affiliations:** ^1^Department of Neurology, The Second Affiliated Hospital of Guangzhou University of Chinese Medicine, Guangzhou 510120, China; ^2^Department of Neurology, Guangdong Provincial Hospital of Chinese Medicine, Guangzhou 510120, China; ^3^College of Basic Medicine, Guangzhou University of Chinese Medicine, Guangzhou 510006, China; ^4^Science and Technology Innovation Center, Guangzhou University of Chinese Medicine, Guangzhou 510405, China

## Abstract

Vascular dementia (VaD), the second cause of dementia, is caused by chronic cerebral hypoperfusion, producing progressive damage to cerebral cortex, hippocampus, and white matter. Ligustilide (LIG), one of the main active ingredients of *Angelica sinensis*, exerts the neuroprotective effect on neurodegenerative diseases. However, the mechanism remains unclear. An *in vivo* model of bilateral common carotid artery occlusion and *in vitro* model of oxygen glucose deprivation (OGD) were employed in this study. LIG (20 or 40 mg/kg/day) was intragastrically administered to the VaD rats for four weeks. The results of the Morris water maze test demonstrated that LIG effectively ameliorated learning and memory deficiency in VaD rats. LIG obviously relieved neuronal oxidative stress damage by increasing the activities of catalase (CAT), superoxide dismutase (SOD), and glutathione peroxidase (GSH-PX) and decreasing the level of malondialdehyde (MDA) in VaD rats. Nissl staining showed that LIG increased the number of the Nissl body in VaD rats. After LIG administration, the apoptotic-related protein, Bax, was decreased and Bcl-2 was increased in the hippocampus of VaD rats. Moreover, the expressions of sirtuin 1 (SIRT1) and protein disulfide isomerase (PDI) were decreased, binding immunoglobulin protein (BIP) and phospho-inositol-requiring enzyme-1*α* (P-IRE1*α*), X-box binding protein 1 (XBP1s), and C/EBP-homologous protein (CHOP) were increased in VaD rats. After LIG treatment, these changes were reversed. The immunofluorescence results further showed that LIG upregulated the expression of SIRT1 and downregulated the expression of P-IRE1*α* in VaD rats. In addition, *in vitro* experiment showed that EX-527 (SIRT1 inhibitor) partly abolished the inhibitory effect of LIG on the IRE1*α*/XBP1s/CHOP pathway. In conclusion, these studies indicated that LIG could improve cognitive impairment by regulating the SIRT1/IRE1*α*/XBP1s/CHOP pathway in VaD rats.

## 1. Introduction

Dementia, an irreversible disease, causing a progressive cognitive decline, has become one of the major health problems. Among them, vascular dementia (VaD) is the second cause of dementia after Alzheimer's disease (AD) [1]. Although the pathological mechanism of VaD is not clear, it seems to be related to neurovascular unit destruction, cholinergic hypofunction, oxidative stress, inflammation, blood-brain barrier breakdown, disruption of Zn homeostasis, and so on [2–4].

Due to the high energy requirements, the brain needs a continuous and well-regulated blood supply [5]. Chronic cerebral hypoperfusion causes mitochondrial dysfunction, which leads to a large amount of reactive oxygen species (ROS) accumulation in neurons and activate the endoplasmic reticulum stress (ER stress) pathway [6, 7]. ER stress is to maintain cellular homeostasis within the ER by activating the unfolded protein response (UPR) [8]. However, persistent ER stress activates downstream proteins, such as caspase-12 and CHOP, which promote cell apoptosis in neuron [9]. Some studies have shown that ER stress occurred in the brain of VaD rats, and inhibiting the ER stress pathway could improve their cognitive impairment [10, 11] .

Sirtuin1 (SIRT1), a NAD+-dependent deacetylase, is thought to participate in the regulation of cellular senescence and aging [12]. Some studies have shown that increasing the expression of SIRT1 could improve cognitive impairment in VaD rats by promoting angiogenesis, anti-inflammatory, antioxidant, and antiapoptosis [13–15]. Interestingly, recent studies have reported that the activation of SIRT1 could alleviate cell damage caused by ER stress [16, 17]. Besides, our previous study also indicated that SIRT1 could downregulate the ER stress pathway to exert a neuroprotective effect in neurodegeneration disease [18].

*Angelica sinensis*, a commonly used traditional Chinese medicine, is well known for its hematopoietic, antioxidant, and immunoregulatory effect [19]. Ligustilide (LIG), one of the main active ingredients of *Angelica sinensis*, has been reported that it can cross the blood-brain barrier [20]. LIG has been reported to have a number of beneficial effects, such as anti-inflammatory [21], antioxidant [22], antiapoptotic [23], angiogenesis-promoting [24], and anticancer [25] effects. Studies have shown that LIG could improve cognitive impairment and protect against neuron damage in VaD rats [26–28]. Besides, our previous studies have shown that LIG can exert a neuroprotective effect in aging mice [29]. However, whether the SIRT1/ER stress pathway participated in the protective effect of LIG on VaD is still unclear.

In this study, an *in vivo* model of bilateral common carotid artery occlusion and *in vitro* model of oxygen glucose deprivation (OGD) were employed to explore the neuroprotective effect of LIG in VaD and its potential mechanism. We found that LIG could reduce 5neuronal damage and improve cognitive impairment by regulating the SIRT1/IRE1*α*/XBP1s/CHOP pathway.

## 2. Material and Method

### 2.1. Material

Ligustilide (CAS: 4431-01-0, purity ≥ 98%, molecular weight: 190.24) was purchased from Chengdu Refines Biotechnology Company (Chengdu, China). Isoflurane was purchased from RWD Life Science (Shenzhen, China). Kit for detecting malondialdehyde (MDA, CAS: A003-1-2), superoxide dismutase (SOD, CAS: A001-1-2), catalase (CAT, CAS: A007-1-1), glutathione peroxidase (GSH-PX, CAS: A005-1-1), and Nissl staining were purchased from Nanjing Jiancheng Bioengineering Institute. PBS, RIPA (CAS: P0013B), bicinchoninic acid (BCA, CAS: P0012S) protein assay kit, and DAPI staining solution (CAS: P0131) were purchased from Beyotime Biotechnology. Dimethyl sulfoxide (DMSO) was purchased from MP Biomedicals, LIC. Dulbecco's modified Eagle's medium (DMEM), fetal bovine serum (FBS), and penicillin/streptomycin were purchased from Gibco Invitrogen Corporation. MTT (CAS: 57360-69-7) was obtained from Sigma-Aldrich. EX527 (CAS: 49843-98-3) was purchased from MedChemExpress. Primary antibodies of Bax (CAS: 2772s), Bcl2 (CAS: 7498s), sirtuin-1 (SIRT1, CAS: 9475s), binding immunoglobulin protein (BIP, CAS: 3177s), protein disulfide isomerase (PDI, CAS: 3501s), protein kinase R-like endoplasmic reticulum kinase (PERK, CAS: 3192s), phospho-protein kinase R-like endoplasmic reticulum kinase (P-PERK, CAS: 3179s), inositol-requiring enzyme-1*α* (IRE1*α*, CAS: 3294s), activating transcription factor 6 (ATF6 CAS: 65880s), and C/EBP-homologous protein (CHOP, CAS: 2895) were purchased from Cell Signaling Technology, Inc. Primary antibodies of *β*-actin (CAS: ab8227) and X-box binding protein 1 (XBP1, CAS: ab37152) were purchased from Abcam, Inc. The primary antibody phospho-inositol-requiring enzyme-1*α* (P-IRE1*α*, CAS: NB100-2323) was purchased from Novus Biologicals. Secondary antibodies (goat anti-mouse IgG, CAS: S0002; goat anti-rabbit IgG, CAS: S0001) were from Affinity Biosciences. A secondary antibody (anti-rabbit IgG (H+L), CAS: 4413s) was from Cell Signaling Technology, Inc.

### 2.2. Animal and Surgical Procedure

Male Sprague-Dawley rats (*n* = 48; weight, 270-280 g) were supplied by the Experimental Animal Centre of Guangzhou University of Chinese Medicine (Guangzhou, China). All rats were raised in a SPF room and were given free access to food and water. After one week of adaptive housing and feeding, we choose twelve rats randomly to receive sham surgery as the control group and thirty-six rats to receive bilateral common carotid artery occlusion surgery as the surgery group. According to previous studies [26, 30], the rats were anesthetized with 4% isoflurane and maintained with 1.5% isoflurane through a vaporizer for isoflurane. Make a middle incision of the neck, and separate the muscle and fascia to expose the common carotid arteries. Then, carefully separate the common carotid arteries from the vagal nerves and cervical sympathetic; after that, permanently ligate the common carotid arteries with 4-0 silk suture. The sham group underwent the same operation without actual ligation. During the whole surgery, all rats' body temperature was maintained at about 37°C.

### 2.3. Animal Drug Administration

LIG was dissolved in a 3% Tween 80 solution. The rats were randomly divided into 4 groups; namely, the sham surgery rats named sham group (*n* = 12) were given equal volume of 3% Tween 80 solution; the surgery rats were randomly divided into 3 groups, namely, the VaD group (*n* = 12, 3% Tween 80 solution), the LIG low-dose group (*n* = 12, 20 mg/kg/d), and the LIG high-dose group (*n* = 12, 40 mg/kg/d).

### 2.4. Morris Water Maze Test

After four-week drug treatment, we used the Morris water maze test to assess the spatial learning memory capacities of the rats as previous studies [26]. The equipment of MWM is composed of a black circular pool, an escape platform, and recording system. The circular pool was filled with nontoxic black coloring water (23 ± 1°C) and was divided into four equal quadrants. Place the escape platform 2 cm below the water surface in the center of the target quadrant. In the hidden platform trials, the rats were released from four different positions per day and last for five days. The swimming path and the time (escape latency) of finding the hidden platform were recorded. Once the rats were released into water, the recording system started and stopped when the rat found the platform and stayed for 3 s or stopped at 60 s when the rats failed to find the platform within 60 s. After the end, we guided the rats to the platform and stayed for 10 s. For the 6th day, the escape platform was removed and released the rats in a new position. Then, the rats swam freely for 60 s. The time spent on the target quadrant and crossing times of the original platform location and the swimming speed of the rats were recorded.

### 2.5. Brain Sections and Tissue Preparation

After the behavior test, rats were anesthetized intraperitoneally with 1% sodium pentobarbital (40 mg/kg) and euthanized by cervical dislocation. The brain tissues of some rats were rapidly removed, and the cerebral cortex and hippocampus were separated from the brains. Then, tissues were quickly stored at -80°C for detecting western blot or kits subsequently. For the microscope observation, rats were perfused with PBS and 4% paraformaldehyde after being anesthetized, and the brain tissue was quickly separated and soaked in 4% paraformaldehyde solution for 24 h. Then, it was embedded in paraffin, and 3 mm thickness sections were made.

### 2.6. Oxidative Stress Level Test

Take an appropriate amount of brain tissue, and place them into ice-cold saline; the brain tissue was homogenized and centrifuged at 12,000 × *g* for 10 min at 4°C. Collect the supernatant to measure the protein concentration by the BCA method. Then, detect the levels of GSH-PX, CAT, SOD, and MDA according to the corresponding reagent instructions. The absorbance was measured at a wavelength of 412, 405, 550, and 532 nm, respectively.

### 2.7. Nissl Staining

The paraffin sections of the rats were deparaffinized and rehydrated and washed with PBS and underwent Nissl staining for 10 min at 37°C. Then, wash the slices with distilled water. Images were captured using a light microscope (Leica Microsystems, Inc.).

### 2.8. Immunofluorescence

The paraffin sections of the rats were deparaffinized and rehydrated. Place the sections into the sodium citrate buffer, and heat them with microwave for antigen retrieval; the sections were incubated with 3% H_2_O_2_ for 10 minutes, washed with PBS, and infiltrated with 0.1% Triton-100 for 20 minutes. Then, the sections were blocked with 5% BSA. Afterwards, the sections were incubated with primary antibodies SIRT1 and P-IRE1*α* at 4°C overnight. Incubate the sections with the fluorescent-conjugated secondary antibody anti-rabbit IgG at 37°C for 2 h. After washing, the sections were incubated with DAPI staining solution. We used a laser scanning confocal microscope (ZESS 8.0, Germany) to detect fluorescence. ImageJ 8.0 (National Institutes of Health) image analysis software is used for image analysis.

### 2.9. Cell Culture and Grouping

PC12 cell lines were obtained from the Cell Bank of Shanghai Institute of Biochemistry (Shanghai, China). PC12 cells were cultured in complete medium, which is composed of 89% DMEM, 1% penicillin/streptomycin, and 10% FBS. And they were cultured in an incubator with a stable environment (5% CO_2_, 37°C). Change the medium every 2-3 days, and the cells were grouped and experimented after three generations. The cells were divided into five groups: control, control+LIG, OGD, OGD+LIG, and OGDZ+LIG+EX527. The addition of the SIRT1 inhibitor EX527 was used to evaluate whether there is a direct link between SIRT1 and the inhibitory effect of LIG on ER stress.

### 2.10. OGD Model Establishment

PC12 cells were grown to a density of 60-70% in a normal incubator. Then, replace the medium with Earle's balanced salt solution, and cells were incubated in a three-gas incubator with a hypoxic environment (94% N_2_, 5% CO_2_, and 1% O_2_, 37°C) for 2 h.

### 2.11. Cell Drug Administration

The cellular administration method of LIG is dissolved in DMSO at a concentration of 0.5 M as a stock solution and stored at -20°C. The SIRT1 inhibitor EX527 was dissolved in DMSO at a concentration of 0.01 M as a stock solution and stored at -20°C. Dilute the stock solution of LIG and EX527 with fresh medium to the final concentration. The final DMSO concentration did not exceed 0.1%, which is nontoxic to cells. In the control+LIG group, the cells were treated with LIG (80 *μ*M) for 2 h. In the OGD+LIG group, the cells were treated with LIG (80 *μ*M) and then subjected to OGD for 2 h. In the OGD + LIG + EX527 group, the cells were pretreated with EX527 for 24 h and then treated with LIG (80 *μ*M) and EX527 (10 *μ*M) together and subjected to OGD for 2 h.

### 2.12. Cell Viability Assay

Seed the cells in 96-well plates at a density of 5000 cells per well. After culturing for 24 hours, the cells were processed accordingly, and then, the cell viability was measured by the MTT method. Prepare a 10% MTT solution with fresh medium, add it to the 96-well plates, and incubate it for another 4 hours. After removing the medium, add 200 *μ*l DMSO into each well to dissolve the formazan crystals. Measure the absorbance of the cells at 570 nm, and compare the results with the control group and the OGD group.

### 2.13. Western Blot Analysis

The hippocampal tissues of the rats and PC12 cells were made into ice-cold RIPA lysis solution for 10 min and 30 min, respectively. The brain tissues were homogenized at 4°C and centrifuged at 12,000 × *g* for 10 min at 4°C. The supernatants were collected to determine the total protein concentration via the BCA method. After that, add appropriate volume of loading buffer and boil it for 10 min at 100°C. Separate protein samples (30-50 *μ*g per well) with 8, 10, or 12% SDS-polyacrylamide gels, and transfer the protein to a polyvinylidene fluoride (PVDF) membrane. Block the membranes with 5% skimmed milk for 2 h at 37°C. Wash the membranes 3 times and incubate them with the primary antibody (Bcl2, Bax, SIRT1, BIP, PDI, PERK, P-PERK, IRE1*α*, P-IER1*α*, ATF6, XBP1s, CHOP, and *β*-actin) at 4°C overnight. Then, incubated them with goat anti-rabbit IgG or goat anti-mouse IgG for 1.5 h. Blot digital images were visualized with an Image Lab 3.0 (Bio-Rad).

### 2.14. Statistical Analysis

The experimental data were presented as mean ± SEM. SPSS 24.0 software (SPSS, Inc.) was used for data analysis, and GraphPad Prism 8 software (GraphPad, Inc.) was used for data processing. The ANOVA (one-way ANOVA or two-way ANOVA) was used to analyze statistical differences in data between groups followed by Dunnett's post hoc test. *P* < 0.05 was considered to indicate statistical significance.

## 3. Results

### 3.1. LIG Improves Learning and Memory Deficiency in VaD Rats

**Figure 1:**
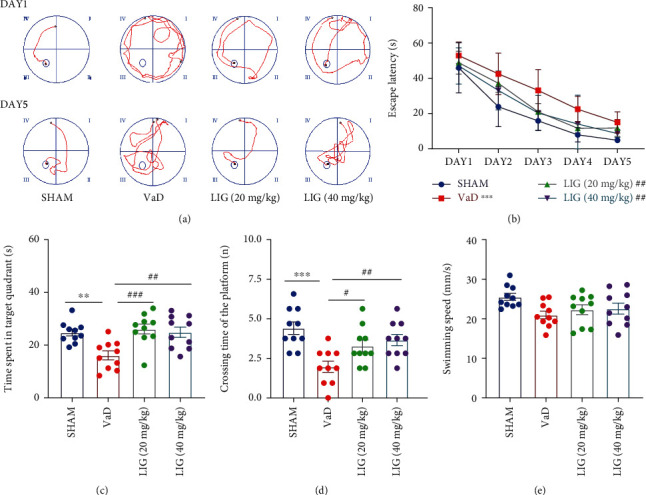
LIG improves learning and memory deficiency in VaD rats. (a) Swimming tracks of rats on the first and fifth day in the Morris water maze test. (b) Escape latency from five consecutive days of the Morris water maze test. (c) Time spent in the target quadrant in the Morris water maze test. (d) Crossing times of the target platform in the Morris water maze test. (e) Swimming speed of rats in the Morris water maze test. Data represent mean ± SEM (*n* = 10 per group). ^∗^*P* < 0.05, ^∗∗^*P* < 0.01, and ^∗∗∗^*P* < 0.001 vs. sham group; ^#^*P* < 0.05, ^##^*P* < 0.01, and ^###^*P* < 0.001 vs. VaD group.

As shown in Figures 1(a) and 1(b), on the five-day hidden platform trial of Morris water maze, VaD rats showed disordered swimming paths, compared to sham group rats (Figure 1(a)). The escape latency was obviously decreased during the five consecutive days. Compared to the sham group rats, VaD rats needed more time to find the hidden platform. LIG treatment (20 or 40 mg/kg) shortened the escape latency, compared to the VaD rats (Figure 1(b)). On the 6th day, the time spent in the original platform and the number of times crossing over the original position of the platform were significantly reduced in VaD rats. As expected, these defects were ameliorated by LIG treatment. However, there is no significant difference in the swimming speed among these groups (Figures 1(c) and 1(e)). Thus, these data demonstrated that LIG could improve learning and memory deficiency in VaD rats.

### 3.2. LIG Reduces Neurodegeneration in VaD Rats

**Figure 2:**
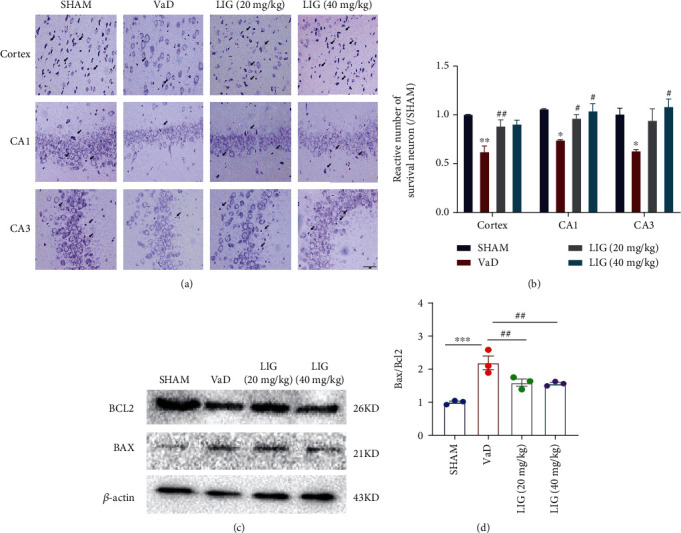
LIG reduces neurodegeneration in VaD rats. (a, b) Nissl staining of the cortex and the hippocampus. Scale bar: 50 *μ*m. (c, d) Western blots of BCL2 and BAX. Data represent mean ± SEM (*n* = 3 per group). ^∗^*P* < 0.05, ^∗∗^*P* < 0.01, and ^∗∗∗^*P* < 0.001 vs. sham group; ^#^*P* < 0.05, ^##^*P* < 0.01, and ^###^*P* < 0.001 vs. VaD group.

We next examined the neuronal degeneration and apoptosis in the brain of VaD rats. Nissl body was weakly stained and lost in the cerebral cortex and hippocampus of VaD rats, compared to the sham group. After LIG treatment, Nissl bodies were stained deeper and increased in numbers (Figures 2(a) and 2(b)), which indicated that LIG could reduce neurodegeneration. In addition, the expression of anti-apoptotic protein, Bcl-2, was reduced, while the proapoptotic protein, Bax, was increased in the hippocampus of the VaD group (Figures 2(c) and 2(d)). LIG increased the expression of Bcl-2 and decreased the expression of Bax. Therefore, these data indicated that LIG could reduce neurodegeneration in VaD rats.

### 3.3. LIG Decreases Oxidative Stress in the Brain of VaD Rats

**Figure 3:**
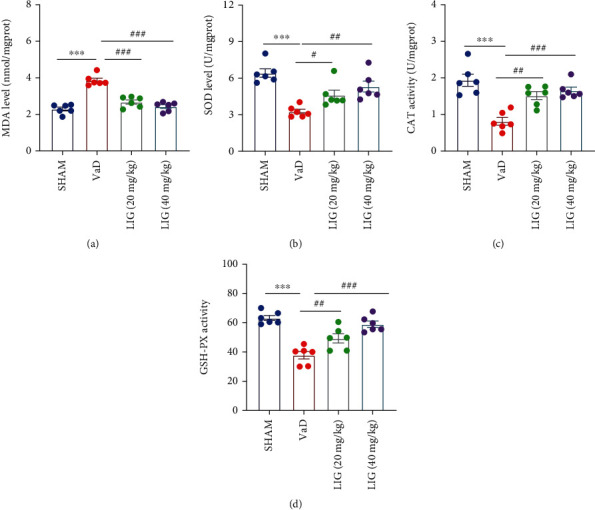
LIG decreases oxidative stress in the brain of VaD rats. (a) The level of MDA. (b) The activity of SOD. (c) The activity of CAT. (d) The activity of GSH-PX. Data represent mean ± SEM (*n* = 6 per group). ^∗^*P* < 0.05, ^∗∗^*P* < 0.01, and ^∗∗∗^*P* < 0.001 vs. sham group; ^#^*P* < 0.05, ^##^*P* < 0.01, and ^###^*P* < 0.001 vs. VaD group.

We further evaluated the effect of LIG on oxidative stress. Compared to sham group rats, the level of MDA increased significantly, while the contents of CAT, SOD, and GSH-PX decreased significantly in the brain of VaD rats, which indicated that oxidative stress happened in VaD rats. After administration of LIG, the level of MDA was decreased and the contents of CAT, SOD, and GSH-PX were increased (Figures 3(a)–3(d)). These results indicated that LIG could decrease oxidative stress in the brain of VaD rats.

### 3.4. LIG Activates SIRT1 and Relieves ER Stress in the Hippocampus of VaD Rats

**Figure 4:**
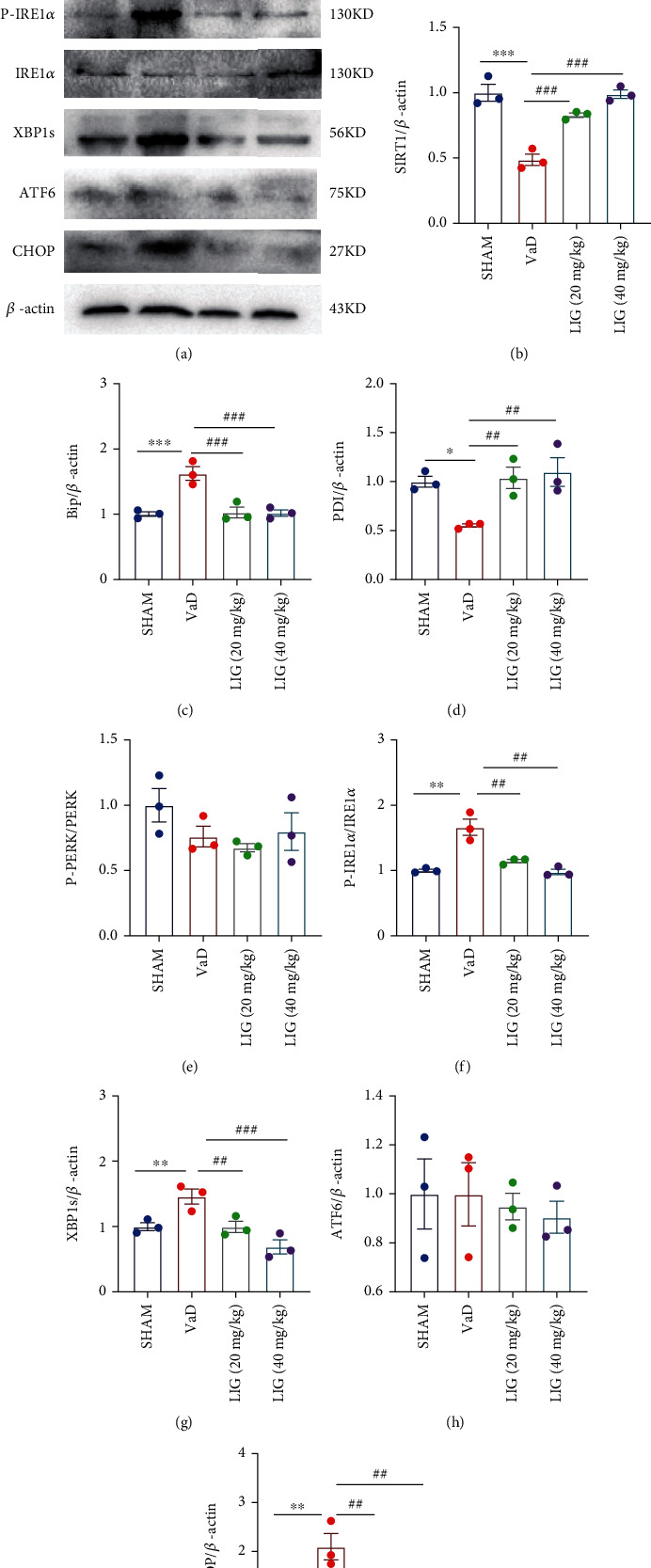
LIG activates SIRT1 and relieves ER stress in the hippocampus of VaD rats. (a–i) Western blots of SIRT1, BIP, PDI, P-PERK, PERK, P-IRE1*α*, IRE1*α*, XBP1s, ATF6, and CHOP. Data represent mean ± SEM (*n* = 3 per group). ^∗^*P* < 0.05, ^∗∗^*P* < 0.01, and ^∗∗∗^*P* < 0.001 vs. sham group; ^#^*P* < 0.05, ^##^*P* < 0.01, and ^###^*P* < 0.001 vs. VaD group.

**Figure 5:**
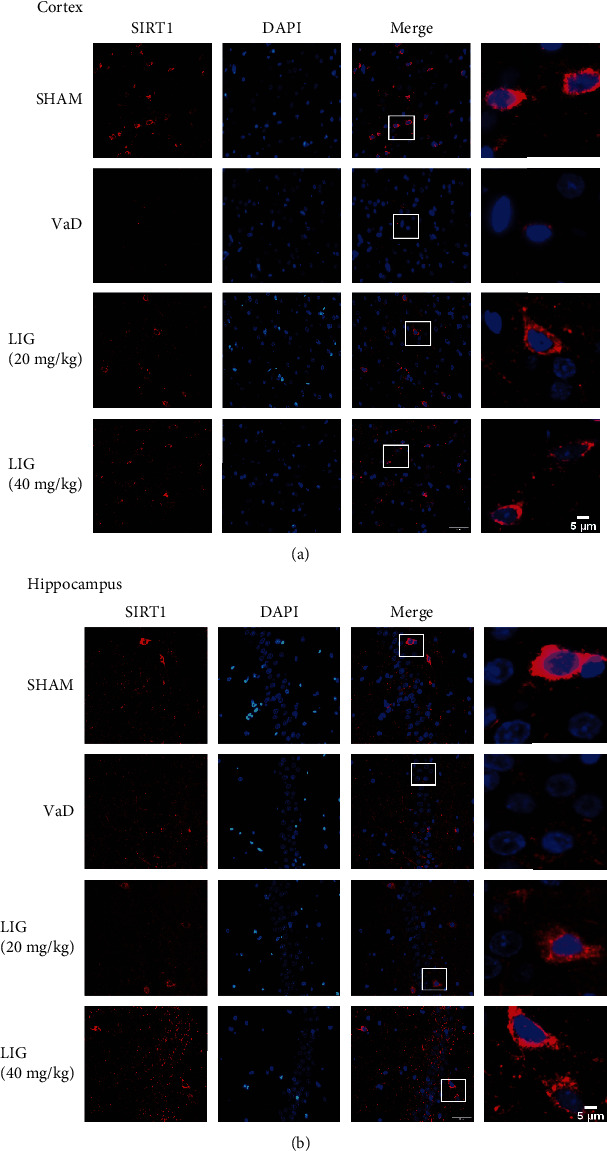
LIG activates SIRT1 in VaD rats. (a) Immunofluorescence of SIRT1 in the cortex (*n* = 3 per group). Scale bar: 50 *μ*m and 5 *μ*m. (b) Immunofluorescence of SIRT1 in the hippocampus (*n* = 3 per group). Scale bar: 50 *μ*m and 5 *μ*m.

**Figure 6:**
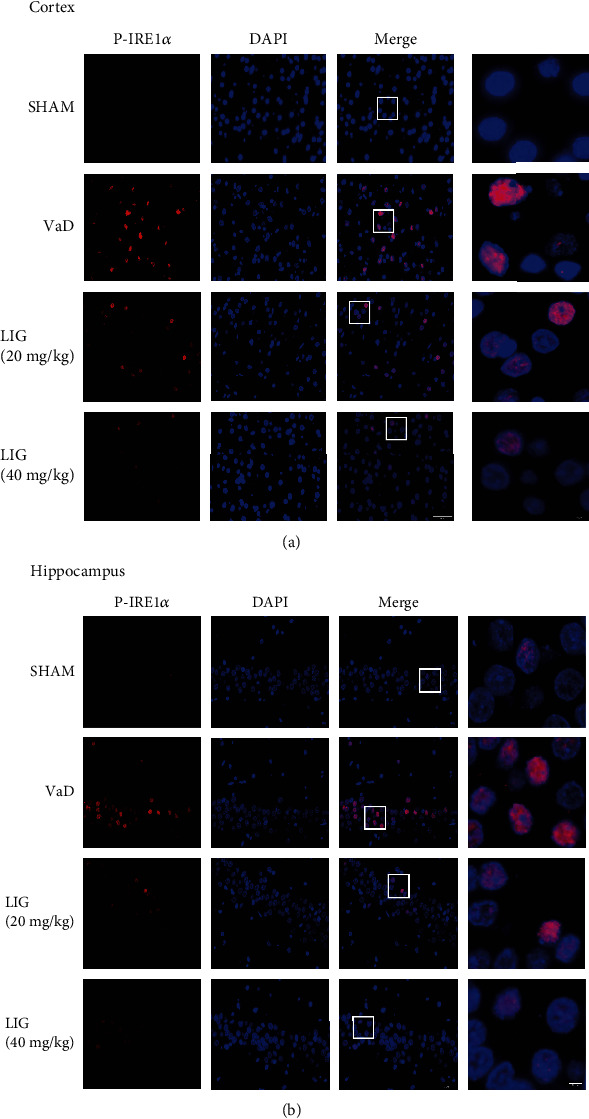
LIG reduces the expression of P-IRE1*α* in VaD rats. (a) Immunofluorescence of P-IRE1*α* in the cortex (*n* = 3 per group). Scale bar: 50 *μ*m and 5 *μ*m. (b) Immunofluorescence of P-IRE1*α* in the hippocampus (*n* = 3 per group). Scale bar: 50 *μ*m and 5 *μ*m.

To explore whether the SIRT1/ER stress pathway is participated in the neuroprotective effect of LIG on VaD rats, SIRT1 and ER stress-related proteins were detected in the hippocampus of VaD rats. In both western blot (Figures 4(a) and 4(b)) and immunofluorescence (Figures 5(a) and 5(b)), results showed that LIG upregulated SIRT1 in VaD rats. The ER stress-related proteins (BIP, P-IRE1*α*, XBP1s, and CHOP) significantly increased and PDI decreased in VaD rats. After LIG treatment, the expressions of BIP, P-IRE1*α*, XBP1s, and CHOP drastically reduced and PDI increased compared to the VaD group (Figures 4(a), 4(c), 4(d), 4(f), 4(g), 4(i), 6(a), and 6(b)). The expression of other ER stress-related proteins (P-PERK, ATF6) showed no significant difference among these groups (Figures 4(a), 4(e), and 4(h)). Thus, these data indicated that LIG could activate SIRT1 and relieve ER stress (IRE1*α*/XBP1s/CHOP pathway) in the hippocampus of VaD rats.

### 3.5. LIG Downregulates the IRE1*α*/XBP1s/CHOP Pathway by Activating SIRT1 in PC12 Cells

**Figure 7:**
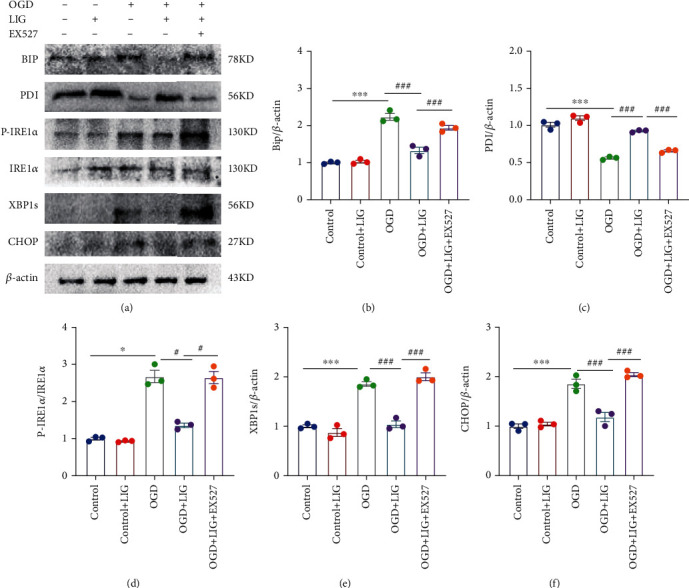
LIG downregulates the IRE1*α*/XBP1s pathway by activating SIRT1 in PC12 cells. (a–f) Western blots of BIP, PDI, P-IRE1*α*, IRE1*α*, XBP1s, and CHOP. Data represent mean ± SEM (*n* = 3 per group). ^∗^*P* < 0.05, ^∗∗^*P* < 0.01, and ^∗∗∗^*P* < 0.001 vs. control group; ^#^*P* < 0.05, ^##^*P* < 0.01, and ^###^*P* < 0.001 vs. OGD+LIG group.

In order to determine whether the expression of SIRT1 is related to the effect of LIG in relieving ER stress, we used an in vitro model, OGD-treated PC12 cells for verification. We used EX527, a specific SIRT1 inhibitor, to inhibit SIRT1 activity. MTT results suggested that 2 h OGD and 80 *μ*M LIG were selected for subsequent experiments (Figure S1A-C). As expected, when the expression of SIRT1 was inhibited, the effect of LIG on reducing the expression of the IRE1*α*/XBP1s/CHOP pathway was partly blocked (Figures 7(a)–7(f)). Therefore, these data indicated that LIG downregulates the IRE1*α*/XBP1s/CHOP pathway by activating SIRT1 in PC12 cells.

## 4. Discussion

**Figure 8:**
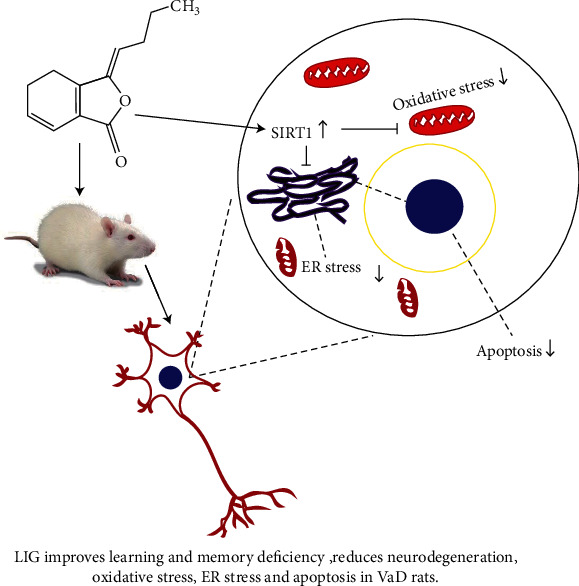
Schematic representation of LIG improves cognitive impairment in vascular dementia rats by regulating the SIRT1/IRE1*α*/XBP1s/CHOP pathway. LIG improves learning and memory deficiency in VaD rats. LIG reduces neurodegeneration, oxidative stress, ER stress, and apoptosis in VaD rats.

In this study, we found that cognitive impairment happened in VaD rats and four-week administration of LIG could improve their learning and memory deficiency. Moreover, LIG could reduce neurodegeneration and relieve neuronal oxidative stress damage in VaD rats. Furthermore, we found that ER stress occurred in the brain of VaD rats. LIG could downregulate the IRE1*α*/XBP1s/CHOP pathway by activating SIRT1 in VaD rats. Therefore, our results proved that LIG could produce neuroprotective effect against VaD, which might be related to the SIRT1/IRE1*α*/XBP1s/CHOP pathway (Figure 8).

Stroke and cardiovascular disease have long been considered the risk of VaD [30]. Unlike AD, which has obvious pathological changes, such as *β*-amyloid and hyperphosphorylated tau deposited in neurons, the biomarkers of vascular dementia are poorly developed [31, 32]. Besides, most genetic researches in dementia were on AD, rather than VaD [33]. Therefore, the pathogenesis of vascular cognitive impairment is still unclear. However, previous studies have suggested that vascular cognitive impairment may be related to white matter lesions and hippocampal atrophy [34–36]. In this study, chronic cerebral hypoperfusion caused cognitive impairment in rats. Oral administration of LIG significantly improved learning and memory deficiency in VaD rats. Therefore, we suggested that LIG could improve learning and memory deficiency in VaD rats.

Antiapoptotic drugs played an important role in attenuating the progression of cognitive impairment in VaD [37]. Previous studies have shown that chronic cerebral hypoperfusion can cause cognitive impairment even though there is no overt cerebral infarction, which may be related to hippocampal atrophy and neuronal apoptosis [38]. Persistent neuronal apoptosis eventually leads to neurodegeneration [39]. Increasing the expression of Bcl2 can reduce apoptosis, while Bax has the opposite effect [40] . Our study showed that LIG increased the expression of Bcl2 and reduced the expression of Bax. Besides, the results of Nissl staining suggested that LIG could increase the number of Nissl bodies in VaD rats. Thus, these data indicated that LIG could reduce neurodegeneration in VaD rats.

The production of radical species is inevitable and essential because they are produced in normal metabolism [41]. However, uncontrolled free radical production can cause oxidative stress and cell damage, such as DNA damage, inflammation, apoptosis, and necrosis [42]. And it is obvious that oxidative stress is related to the progression of VaD [43]. Endogenous enzymes, such as SOD, CAT, and GSH-PX, can effectively scavenge free radicals to against oxidative stress damage [43]. MDA, an index of lipid per oxidation, can indirectly reflect the level of oxygen free radicals in cells [44]. In this study, MDA was increased, while SOD, GSH-PX, and CAT were decreased in VaD rats, which indicated that oxidative stress occurred in VaD rats. LIG treatment reversed these changes. Therefore, these data suggested that LIG could decrease the level of oxidative stress in the brain of VaD rats.

SIRT1 plays a crucial role in neurodegeneration disease [45]. It can reduce neuronal loss, suppress inflammation, and inhibit apoptosis and oxidative stress in the hippocampus of VaD rats [46–48]. Previous studies have shown that chronic cerebral hypoperfusion leads to a downregulation of SIRT1 in the brain of VaD rats, which is similar to our study [15, 47]. However, oral administration of LIG enhanced the expression of SIRT1 in the cortex and the hippocampus of VaD rats. ER stress is highly associated with learning and memory deficiency in both vascular dementia and AD [11, 49]. When UPR occurred, three pathways of ER stress, PERK, IRE1*α*, and ATF6, are activated to maintain cell homeostasis. However, when UPR exceeds the load of the ER, activating transcription factor 4 (ATF4), XBP-1, and ATF6 are transferred to the nucleus to activate the CHOP protein leading to neuronal apoptosis [50–52]. Moreover, some studies have proven that SIRT1 could suppress ER stress in different diseases [17, 53, 54]. SIRT1 can deacetylate eif2*α* to reduce the damage cause by ER stress in cardiomyocytes [16]. In this study, ER stress was markedly activated in VaD rats. LIG inhibited the activation of the IRE1*α*/XBP1s/CHOP pathway. Besides, to determine whether there is a direct link between SIRT1 and the inhibitory effect of LIG on IRE1*α*/XBP1s/CHOP pathway activation in VaD, we used EX527, an inhibitor of SIRT1, for verification. Results showed that SIRT1 suppression partly abolished the inhibitory effect of LIG on the IRE1*α*/XBP1s/CHOP pathway *in vitro*, which demonstrated that LIG inhibited the activation of IRE1*α*/XBP1s/CHOP by activating SIRT1. Although our research found that LIG could upregulate SIRT1, we do not know whether LIG could act as the effect as resveratrol, a common activator of SIRT1. Therefore, in further studies we will employ resveratrol as the positive drug for LIG in the VaD model.

In conclusion, the neuroprotective effect of LIG against VaD was proven in VaD rats. Besides, it was found that LIG improved cognitive impairment by regulating the SIRT1/IRE1*α*/XBP1s/CHOP pathway in the hippocampus of VaD rats. However, the further mechanisms are still needed to be explored.

## Data Availability

The datasets used and/or analyzed during the current study are available from the corresponding author on reasonable request.

## Supplementary Materials

Supplementary Materials Supplementary Figure 1: protective effects of LIG on OGD-treated PC12 cells.
