# Supplementary material for: Ligustilide Improves Cognitive Impairment via Regulating the SIRT1/IRE1α/XBP1s/CHOP Pathway in Vascular Dementia Rats
Source: Oxid Med Cell Longev. 2022 Aug 16;2022:6664990. doi: 10.1155/2022/6664990 (PMC9398841; doi:10.1155/2022/6664990)
Supplement: Supplementary Materials — Supplementary Figure 1: protective effects of LIG on OGD-treated PC12 cells. [file 6664990.f1.docx]

**Supplementary Figure 1**


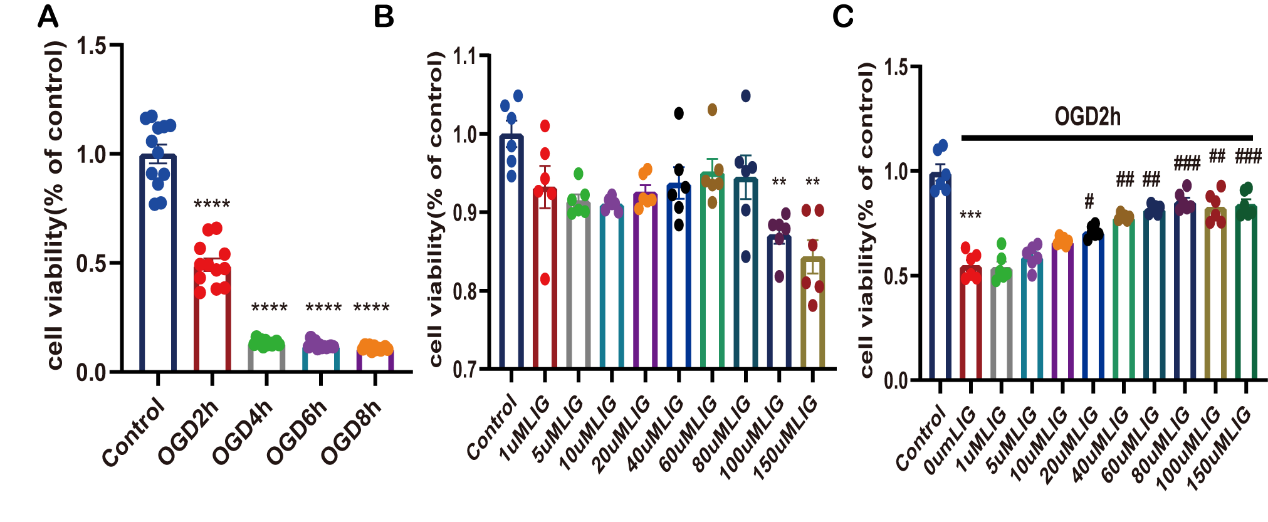


**Supplementary Figure 1 Protective effects of LIG on OGD-treated PC12 cells.** (A) PC12 cells were treat with OGD for 2h, 4h, 6h, 8h. (B) PC12 cells were treat with 1μM,5μM,10μM,20μM,40μM,60μM,80μM,100μM,150μM for 2h. (C) PC12 cells were treat with 1μM,5μM,10μM,20μM,40μM,60μM,80μM,100μM,150μM and OGD for 2h. Data represent mean ± SEM (n = 6-12 per group). * *P* < 0.05, ** *P* < 0.01, *** *P* < 0.001 vs. Control group; # *P* < 0.05, ## *P* < 0.01, ### *P* < 0.001vs. OGD group.
